# Systematic Review of Capnography with Mask Ventilation during Cardiopulmonary Resuscitation Maneuvers

**DOI:** 10.3390/jcm8030358

**Published:** 2019-03-13

**Authors:** Francisco José Cereceda-Sánchez, Jesús Molina-Mula

**Affiliations:** 1 Emergency Care Management 061, Balearic Islands, C/. Illes Balears s/n., 07014 Palma de Mallorca, Spain; 2 Physiotherapy Department at the University of Balearic Islands, Ctra. de Valldemossa, km 7.5, 07122 Palma de Mallorca, Balearic Islands, Spain; jesus.molina@uib.es

**Keywords:** bag-valve-mask, capnography, cardiopulmonary resuscitation, heart arrest, non-invasive ventilation

## Abstract

The latest guidelines identify capnography as an instrument used to assess bag-valve-mask ventilation during cardiopulmonary resuscitation (CPR). In this review, we analyzed the feasibility and reliability of capnography use with face mask ventilation during CPR maneuvers in adults and children. This systematic review was completed in December 2018; data for the study were obtained from the following databases: EBSCOhost, SCOPUS, PubMed, Índice Bibliográfico Español en Ciencias de la Salud (IBECS), TESEO, and Cochrane Library Plus. Two reviewers independently assessed the eligibility of the articles; we analyzed publications from different sources and identified studies that focused on the use of capnography with a face mask during CPR maneuvers in order to describe the capnometry value and its correlation with resuscitation outcomes and the assistance of professionals. A total of 888 papers were collected, and 17 papers were included that provided objective values for the use of capnography with a mask for ventilation. Four were randomized clinical trials (RCT) and the rest were observational studies. Four studies were completed in adults and 13 were completed in newborns. After the analysis of the papers, we recommended a capnographic level of C in adults and B in newborns. Despite the little evidence obtained, capnography has been demonstrated to facilitate the advanced clinical practice of mask ventilation in cardiopulmonary resuscitation, to be reliable in the early detection of heart rate increase in newborns, and to asses in-airway patency and lung aeration during newborn resuscitation.

## 1. Introduction

Myocardial infarction is the major cause of death worldwide in a certain age group according to the most current global disease-specific mortality data [1]. The initial airway management support provided by medical staff to patients with cardiac arrest is usually a bag-valve-mask (BVM) in cardiopulmonary resuscitation (CPR) [2,3]. There is still controversy about the best device to use for airway management during CPR [4]. Historically, orotracheal intubation (OTI) was the recommended golden standard [5,6]; the latest recommendations of the International Liaison Committee on Resuscitation (ILCOR) has doubted whether there are any superior advanced airway management devices that can be compared to BVM and that provide better results after a cardiac arrest, as supported by several observational studies [4].

Capnography is the non-invasive monitoring of ventilation via carbon dioxide (CO_2_) present in exhaled gas with a waveform representation, while capnometry is the value alone without waveform [7,8]; it complements the monitoring of a patients’ oxygenation by means of pulse-oximetry [9]. Capnography is usually expressed as the partial pressure (normal levels are 35 to 40 mmHg) of the maximum CO_2_ level obtained at the end of expiration (better known as end-tidal CO_2_ or EtCO_2_) [10]. The technological advances of the last decades have produced evolved instruments for monitoring this parameter with increasingly precise and compacted instruments. Now capnographs are even incorporated in defibrillator monitors used in emergency services [9] as well as in respiratory function monitors in delivery rooms [11]. They are compact devices with a portable size.

Different technologies are used for monitoring EtCO_2_: (1) Colorimetric detectors, which are simple devices that react chemically with CO_2_, providing semi-quantitative values in a three-color range according to the existing CO_2_ concentration in the exhaled gas sample; (2) capnometers represent the measurement as a numerical value of exhaled CO_2_, expressed in pressure units: Torr (mm Hg) or % CO_2_; (3) capnographs determine CO_2_ levels and provide a graphical, waveform representation of the detected values (known as a capnogram) [12], which can be represented by its relation to CO_2_ concentration based on volume (volumetric capnography) or time (temporal capnography). The latter is most commonly used [13].

Scientific societies have been recommending capnography during CPR maneuvers for correct OTI monitoring because it is used for effective and/or quality cardiac massage, and for early detection of the return of spontaneous circulation (ROSC) [4]. The validity of this parameter as a prognostic indicator of the result of cardiac arrest has been tested, correlating the low survival probability of newly intubated patients as those who show values <10 mmHg or the same level after 20 min of reanimation [14].

In the last European Resuscitation Council recommendations, the prominence of capnography in physiological monitoring during cardiac arrest increased. The authors indicated the potential of using this parameter together with complementary ventilation systems (Supraglottic Airway Devices or BVM) for patients experiencing cardiac arrest [15].

The clinical question was: In patients under resuscitation maneuvers (P), (1) is capnographic monitoring during ventilation with a mask (I) feasible? (2) Is it reliable to assess the quality of ventilation techniques and ROSC? (O). The secondary aim of this work was to describe the kinds of instruments used to determine capnometry or capnography and to identify professionals who performed CPR maneuvers with mask ventilation and capnographic monitoring.

## 2. Materials and Methods

This systematic review was completed without an earlier cut-off limit up to 31 December 2018. We followed the steps of the Preferred Reporting Items for Systematic Review and Meta-Analysis protocols (PRISMA) [16], using Health Sciences Descriptors (DeCS/MeSH): Capnography and Cardiopulmonary Resuscitation, and the free terms: End-tidal, EtCO_2_, CPR, and resuscitation. The following combination was established with Boolean operators: (“Capnography” (Mesh) OR “end tidal” OR “etco2”) AND (“Cardiopulmonary Resuscitation” (Mesh) OR “CPR” OR “resuscitation”).

The primary sources of this review were the following databases: EBSCOhost, SCOPUS, PubMed, Índice Bibliográfico Español en Ciencias de la Salud (IBECS), TESEO (Spanish Theses repository), and Cochrane Library Plus. We also looked through the CPR guidelines published since 1998 by ILCOR, the European Resuscitation Council, and the American Heart Association, for papers that referenced the contents of this review.

The inclusion criteria used for the review were: (1) Observational and experimental studies published in English and Spanish, (2) studies should describe the implementation of capnography in CPR maneuvers for patients of any age; (3) studies should assess the effectiveness of ventilation using the mask only or in comparison with other devices for advanced airway management in cardiac arrest patients; and (4) studies should assess the correlation of values obtained from capnography and results of resuscitation (ROSC, survival rates, etc.).

Exclusion criteria included: (1) Studies performed on training mannequins, anesthetized patients, corpses, or animals; (2) clinical cases, letters to the editor, or other editorial texts; and (3) studies only focusing on the use of capnography in intubated patients.

The review was completed in pairs; the final decision as to whether to include or exclude a particular study was agreed upon by the research team, firstly by screening the titles and abstracts and then by a full-text reading. We used a template for the structured assessment of each consulted paper that included the following: Introduction, justification, objectives, kind of study, year of study, sample size, methodology, significant results, discussion, limitations, conclusions, remarks, and recommended bibliography. Then, we evaluated the degree to which each study was adequate on a 4-point Likert scale in terms of the criteria and methodological quality of the results shown. The Likert scale values were as follows: Paper with little relevance to the purpose of our review was assigned 1 point; 2 points when the study was relevant to the theoretical framework of justification of the review, but of low methodological quality; 3 points when the research methodology was relevant, but with results of low interest to the review; and 4 points when the methodology, results, conclusions, and theoretical framework of the work were relevant to the review. For a critical reading of the selected papers, we used the Critical Appraisal Skills Programme (CASP), which is used to complete systematic reviews, available on the web-based application FLC 3.0 [17]. 

After completing the literature research, the strategy was replicated by an expert in Documentation Sciences, using descriptors and their Boolean combination and the language used by databases. The expert obtained the same results. As such, the reliability of our review was ensured.

Both reviewers independently extracted the following data from each paper included in the systematic review and then compared the results obtained by means of a pre-designed chart: Sample size, kind of CPR (cardiac origin, traumatic, adult or pediatric patients, and intra-hospital or out-of-hospital), staff performing CPR, type of capnograph or capnometer used with BVM, physiological or survival values compared by ETCO_2_ (ROSC, survival at hospital admission and discharge, heart rate correlation, and time of measuring), evidence level according to CASP, results of the structured template, and Likert scale.

The methodological quality was evaluated by means of CASP. Based on the results, it was agreed to approximate to the grade recommendations based on the U.S. Preventive Services Task Force (USPSTF) evidence levels from “A” (substantial benefit) to “I” (insufficient evidence) levels.

## 3. Results

**Figure 1:**
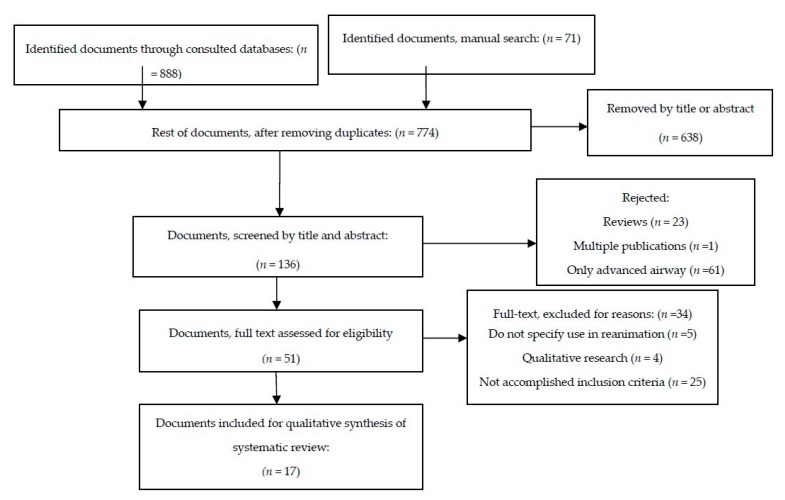
Flow-chart of information according to the review stages.

We reviewed the full text of 136 papers, and 17 were selected after applying the inclusion and exclusion criteria for critical reading (Figure 1). Distribution according to the study design was: eight descriptive prospective (47%), five retrospective (29%), and four RCT (23.5%). 13 were focused on neonatal patients (76.4%) and the other four on adults (23.6%). The mean number of patients in studies focusing on adults was 62, and the mean in newborn studies was 66. All studies on newborns were completed in the delivery room. On adults, three were completed in out-of-hospital cardiac arrest and one intra-hospital cardiac arrest. All the RCT focused on newborns; two of which focused on the viability and reliability of capnography monitoring during mask ventilation. We did not find any study about the use of capnography in children out of the neonatal period.

In adults, the articles relating the feasibility of capnography monitoring during CPR were all observational, and only Nakatani et al. [18] presented data and the correlation between registered values and outcomes from resuscitation, like ROSC or surveillance. In the cluster that used BVM, the ROSC proportion was 15% of the patient groups that showed EtCO_2_ values < 0.5% (<4 mmHg), 14% of patients from the group that showed EtCO_2_ values between 0.5% and 2% (between 4 and 15 mmHg), and 43% of patients from the group with values > 2% (>15 mmHg). The features of studies in adults are summarized in Table 1. Based on these results, the overall recommendation for capnometry use during mask ventilation in adults was level C according to USPSTF.

In newborns, there were more examples of feasibility and reliability capnography monitoring during CPR. From the 13 studies in these patients (four RCT, nine observational), the vast majority considered capnometric values as a parameter for assessing the quality of ventilation (Table 2), airway patency, mask leaks, and showed a good correlation between capnography and heart rates [19,20,21,22,23,24,25,26,27,28,29,30,31]. The two RCTs that emphasized the validity of the technique were: (1) Hawkes et al., who compared the accuracy of colorimetric detector versus capnography to determine normocarbia, but they did not find a difference in the incidence of normocarbia between the two detectors. (2) Kong et al. compared the utility of continuous capnography monitoring versus no capnography monitoring in the delivery room; they did not find a reduction in the occurrence cases in the targeted range (40–60 mmHg) of CO_2_ in the initial blood gas analysis at admission at neither of the two study branches. Although, both studies recommended the use of capnometry as a useful instrument because it provided useful physiologic information about the ventilatory status of the patient. Four articles correlated the use of capnography with assessing the increase in infant heart rate: Palme-Kilander and Tunell [31] appeared to be the first authors to correlate the upper values of exhaled CO_2_ with heart rate increase in newborns. During periods of assisted ventilation with CO_2_ of less than 2 mL/kg/min, they observed a mean of 87 beats/min; with records between 2 and 6 mL/kg/min, they observed an immediate increase in heart rate to 132–159 (mean 145) bpm. Hooper et al. [28] found EtCO_2_ levels increased to >10 mmHg in 28 s (median) before the heart rate increased up to 100 bpm. Mizumoto et al. [25] found an early detection of expired CO2 (>15 mmHg) between 8 and 73 s (median 15 s) before heart rate improvement. Blank et al. [26] indicated that, when the EtCO_2_ value was above 14 mmHg (2% in colorimetric detector), the detector showed an accuracy of 95%, detecting an increase in EtCO_2_ 10 to 20 s before the increase in heart rate appeared on the monitor. They commented that the electrocardiogram did not provide a reliable measurement of heart rate in 12% (5/41) of neonates. 

Considering these studies, we recommended the B level or the use of capnometry detectors during facemask ventilation during newborn resuscitation. 

The staff that assist during resuscitations in pre-hospital environmental studies include paramedics and emergency medical technicians (EMTs), whereas in a hospital environment, medical, midwife, or nursing staff assist.

The limitations of the analyzed studies include the designs used, especially the retrospective design of five primary research papers, due to possible bias during data collection with small-sized samples. Otherwise, in newborn studies, the two RCT with larger samples focused on the values of EtCO_2_ as another variable observed for assessing the quality of ventilation in two different types of BVM [26] and compared the usual positive pressure ventilation through mask and T-piece device with a sustained ventilation technique, where a correlation was found between high EtCO_2_ values and larger volume tidal [25]. The other two RCT focused more on the reliability of capnometry during newborn resuscitation, and reported no improvement or correlation between capnometric values registered and blood PCO_2_ levels. Kong et al. [27] only used the value of capnometry from the last five breaths before disconnection of the positive pressure ventilation and compared this value with PCO_2_ levels with the average from the 40 minutes after EtCO_2_ measurement. Neither aspect of the study produced a reduction in the occurrence of hypocapnia and hypercapnia through capnography monitoring with mask ventilation. Hawkes et al. [20] recognized, as a limitation, a probable alteration in the CO_2_ blood test one hour after registration of the last EtCO_2_ value due to other clinical interventions. Although, both of them indicated that the use of capnography was feasible and appeared to be safe. The vast majority of studies in neonates did not use capnography monitoring as a unique variable or target in the study; the authors used it as a value for assessing the quality of ventilation. Another limitation was the high heterogeneity regarding the patients (four adults and 13 neonate studies), and the kind of cardiac arrest in adults. In the adult studies, one paper included only the cardiac arrest of a non-traumatic origin, another included all kinds of cardiac arrests, and the rest included only cardiac arrest of electromechanical dissociation and/or asystole. Leturiondo et al. [32]. did not describe if the patients were synchronized by chest compressions and ventilation, which obviously would artefact the capnogram. In adults we can say that there was too much variability in the measurement times utilized during the capnography monitoring between the studies.

In general, the instruments for monitoring CO_2_ were different in the studies, providing different ranges, volumetric, time capnography, or colorimetric values. Colorimetric devices were used in five studies; this kind of detector was unable to differentiate inadequate tidal volumes in comparison with the use of respiratory function monitors, which could incorporate CO_2_ monitoring and are used more in the delivery room. 

## 4. Discussion

Within the newborn studies, there was more evidence about the use of capnometry detectors within mask ventilation. A previous review about the use of capnography in the delivery room [11] suggested a level of B for facemask ventilation according to the American Heart Association levels of evidence, which are recommendations that agree with our findings. We did not find any publications that reflected the correlation of quantitative values of capnography during adult CPR maneuvers with resuscitation outcome. Some evidence does exist for the feasibility of the technique in these patients. For example, Pearce et al. [14] assessed the correlation of capnographic values with ROSC as a predictor of hospital discharge. They stated that the capnography placed in OTI or BVM did not provide any more data about values recorded by BVM and capnography. Leturiondo et al. [32] studied the chest compression artifact on capnogram-based ventilation detection and found no distortion in the capnogram of seven patients ventilated with BVM; the rest of the patients were ventilated through OTI or supraglottic airways. They did not provide more capnographic values of resuscitations performed using BVM. Davis et al. [33] showed that capnography was performed immediately after starting ventilation with BVM and later on after OTI. The authors suggested that this approach established an appropriate capnography functioning and allowed for the recording of an EtCO_2_ reference value before intubation. Nakatani et al. [18], in their multicenter study, correlated the values of the CO_2_ detector together with BVM (40% of the cases), laryngeal mask (LMA) (58% of the cases), or esophageal tube (2% of the cases) with variables such as ROSC, survival at hospital admission, and initial electrocardiographic rhythm. They found a significant difference in both the rate of ROSC between patients with EtCO_2_ values of less than 0.5% and those with values greater than 2%. 

We think that the initial parameterization using capnography with BVM for ventilation is crucial in order to provide more objective comparisons with the values obtained by different ventilation devices in CPR and clinical results during reanimation of adults. Although apparently obvious, Kodali and Urman [13] showed that providing ventilation with BVM could result in rather unreliable values due to probable air leaks through the facemask. Ventilation leaks may increase due to inexperience in sealing the interface, through inadequate airway opening, and an increased volume of blown air into the esophagus and subsequently into the stomach. These parameters would be easier to control by incorporating spirometers into the monitors [34], or with volumetric capnography, as has been performed with new-generation monitors in the delivery room and implemented in various newborn studies in order to determine ventilation pressures and volumes, mask leaks, and successful aeration through capnography. As such, capnography monitoring during mask ventilation is feasible and seems reliable in infants without congenital cardiopulmonary abnormalities. Pahuja et al. [19] demonstrated that avoiding high tidal volumes and hypocarbia by means of EtCO_2_ in the delivery room could reduce intraventricular hemorrhage. Finer et al. [30] showed that, with a CO_2_ detector, providers could recognize and attempt to correct airway obstruction. As demonstrated in the literature, newborn CPR used to be enough with mask ventilation and only during the first minutes after transmission from intrauterine to extrauterine life. At this moment, newborns must increase pulmonary blood flow as well as expand fluid-filled alveoli to facilitate gas exchange, and more assistance is required for preterms [11]. This is a physiological situation, which encompasses low flow in minor circulation and asphyxia treatment. Neonate arrest differs from adult arrest, where only artificial blood flow is achieved via compressions once ROSC is achieved. As infant capnography showed reliability in the detection of increase in heart rate, we hypothesized that this quality should be similar for detecting ROSC in adults.

The limitations of our review include the impossibility of performing translations of studies published in languages other than English and Spanish, which reduced the number of papers reviewed. We would like to emphasize the heterogeneity of measurement systems, like the variability in the moment of measurement. Future studies in adults should consider an adequate recording of the times of measurement and including the results obtained, if possible, for up to 20 min, after initiating the maneuvers. Besides the wide variety of professionals who assisted these patients based on the setting (in-hospital or out-of-hospital), country, and respective legislation, the maneuvers can be completed by various kinds of professionals. Trying to control for these variables when comparing of in future studies may help to reduce potential bias. With this many different variables, the meta-analysis of even two papers is unsuitable. 

## 5. Conclusions

Despite having limited and low-level evidence in adults and moderate evidence in newborns (Table 1 and Table 2), according to the evidence levels of the USPSTF and results of the recorded critical reading sheets (FLC 3.0), we suggest that capnography with mask ventilation is feasible and seems to have the potential to advise rescuers about patients in cardiac arrest (adults and neonates). The reliability of the technique has been demonstrated with a correlation observed between capnometric values obtained from a ventilation using mask in those patients, as well as by the usefulness of this parameter for early detection of heart rate increase, sometimes more than 10 s in advance of rate increase, and to help maintain airway patency and lung aeration, which was mainly recorded in neonatal patients. 

## 6. Recommendations for Practice

According to the USPSTF criteria, we recommend a C level for the implementation of capnography in adult mask ventilation during CPR, as evidence quality is low to moderate and net benefit would be small. In newborn resuscitation, we suggest a B level. Capnography can facilitate airway management as an indicator of gas exchange, lung aeration, early identification of obstruction, early recognition of ROSC or heart rate increase, and probably the quality of the administered chest compressions. The obtained results help establish an advanced clinical practice of facemask ventilation based on quantifiable evidence, which is often performed by staff who are not used to and/or not qualified to use more advanced devices; they may be nursing staff, EMTs, or even physicians of primary health care or other specialties not related to emergency and critical care in the pre-hospital setting. 

## Figures and Tables

**Table 1:** Relationship of variables analyzed in the adult studies with evidence levels, and evidence level according to USPSTF.

Author/year	Study Design	Total N/BVM Ventilated N	Location/Cause of Cardiac Arrest	Professional Assisting	Type of Capnography	Outcome	Time of End-tidal CO_2_ Measurement	Evidence Level USPSTF
**Leturiondo et al. 2017**	Observational retrospective	232/7	Out-of-hospital cardiac arrest	Paramedics	Microstream	Distortion of capnogram by chest compression	20 min	III
**Pearce et al. 2015**	Observational retrospective	50/NK	Intra-hospital cardiac arrest by PEA/asystole	A multidisciplinary intra-hospital cardiac arrest team	Mainstream	ROSC and survival at discharge from hospital	Continuous during the first 10 min of cardiac arrest	Not classifiable *
**Davis et al. 2013**	Observational prospective	145/NK	Out-of-hospital cardiac arrest	Paramedics	Did not specify	ROSC and correlation with heart rate values	Before, during, and after pauses to check pulse: every 2 min	Not classifiable *
**Nakatani et al. 1999**	Observational prospective multicentric	121/48	Non-traumatic out-of-hospital cardiac arrest	Emergency technicians	Colorimetric	ROSC, survival at admission	7 to 15 min	II-3

BVM: bag-valve-mask; NK, Not known which are ventilated through the mask; PEA, pulseless electrical activity; ROSC, return of spontaneous circulation; EtCO_2_, end-tidal CO_2_; USPSTF, U.S. Preventive Services Task Force. * according to our clinical question.

**Table 2:** Relationship of variables analyzed in the newborn studies, together with evidence levels and evidence level according to USPSTF.

Author/year	Study Design	Total N/Mask Ventilated	Gestational Age	Professional Assisting	Type of Capnography	Outcome	Time of End-tidal CO_2_ Measurement	Evidence Level USPSTF
**Pahuja et al. 2018**	Observational retrospective	70	Preterm	Clinicians	Mainstream	Incidence of intraventricular hemorrhage and bronchopulmonary dysplasia	Continuous during resuscitation	III
**Hawkes et al. 2017**	Observational prospective	59	Preterm	Physicians	Colorimetric and Microstream	Normocarbia within the first hour of life.	Continuous during delivery room ventilation	I
**Ngan et al. 2017**	Randomized Clinical Trial	162	Preterm	Multidisciplinary delivery team	Mainstream	During sustained inflation or positive pressure ventilation	Continuous, during first 60 s	I
**Thallinger et al. 2017**	Randomized Clinical Trial	328	Term	Midwives and nurse anesthetists	Microstream	Tidal volumes and mask leak. Airway pressures and tidal volume comparing 2 devices	10 min	I
**Hawkes** **et al. 2016**	Observational prospective	35/29	Preterm	Unspecified	Microstream	Feasibility of EtCO_2_ monitoring, normocapnia on admission neonatal intensive care unit	Continuous, during first 10 min	III
**Murthy et al. 2015**	Observational prospective	35/15	Preterm	Unspecified	Mainstream	Effectiveness active inflation, tidal volume and pressures.	During 5 first inflations	III
**Mizumoto et al. 2015**	Observational prospective	15/7	Preterm	Pediatricians and nurses	Mainstream	Increase of heart rate and quality of ventilation	Up to 3 min from delivery.	III
**Blank et al. 2014**	Observational retrospective	41	78% preterm	Multidisciplinary delivery team	Colorimetric	Increase of heart rate	When detector turns to yellow (EtCO_2_ > 15 mmHg)	III
**Kong et al. 2013**	Randomized Clinical Trial	48	Preterm	Multidisciplinary delivery team	Mainstream and colorimetric	Correlation PCO_2_ levels in blood gas	Average from last 5 ventilations	I
**Hooper** **et al. 2013**	Observational prospective	10	Preterm	Multidisciplinary delivery team	Mainstream	Increase of heart rate and quality of ventilation, relationship with tidal volume	Continuous, not specified duration	III
**Murthy et al. 2012**	Observational prospective	40	Preterm	Unspecified	Mainstream	First respiratory effort and tidal volume	Since first inspiratory effort	III
**Finer** **et al. 2009**	Observational retrospective	24	Preterm	Multidisciplinary delivery team	Colorimetric	Determining if airway was patent	Continuous, not specified duration	III
**Palme-Kilander and Turner. 1993**	Observational prospective	30/28	Term	Unspecified	Beckman Liston Becker II (volumetric)	Increase of heart rate and quality of ventilation	Every 15 s. Up to 5 min	III

NK, Not known which are ventilated through the mask; PEA, pulseless electrical activity; ROSC, return of spontaneous circulation; EtCO_2_, end-tidal CO_2_; USPSTF, U.S. Preventive Services Task Force.
